# Repurposing China’s industrial solid waste in mine ecological restoration

**DOI:** 10.1016/j.xinn.2026.101567

**Published:** 2026-09-08

**Authors:** Qiuxia Zou, Ning Zhang, Zeguo Yang, Xiong Shi, Huabo Duan, Qifei Huang, Jiakuan Yang

**Affiliations:** 1 Institute of Artificial Intelligence, School of Artificial Intelligence and Automation, Huazhong University of Science and Technology, Wuhan 430074, China; 2 Hubei Key Laboratory of Multi-media Pollution Cooperative Control in Yangtze Basin, School of Environmental Science & Engineering, Huazhong University of Science and Technology, Wuhan 430074, China; 3 Department of Civil and Environmental Engineering, University of California, Davis, Davis, CA 95616, USA; 4 National Engineering Research Center of Eco-Environment in the Yangtze River Economic Belt, Wuhan 430014, China; 5 State Environmental Protection Key Laboratory of Hazardous Waste Identification and Risk Control, Chinese Research Academy of Environmental Sciences, Beijing 100012, China

## Main text

Across the globe, non-hazardous general industrial solid waste (ISW) from mining, manufacturing, energy production, and other sectors is piling up. In 2022, global ISW generation exceeded 16 billion tonnes (Bt), and China accounted for about one-quarter of the total (Figure 1A). The pressure from bulk ISW is particularly severe in China, where more than 32 Bt remains in storage or long-term stockpiles (Figures 1B and 1C). Meanwhile, traditional recovery as building materials has weakened because market demand has contracted, while new landfills or stockpiles face land scarcity and tighter environmental controls. To tackle the dual challenges of bulk ISW stockpiles and stagnant utilization in construction, China is therefore turning to mine backfilling and pit filling. In January 2026, China’s State Council released the Action Plan for Comprehensive Solid Waste Management (known as “Ten Actions” for solid waste),^1^ establishing a governance framework for managing mine-waste backfilling and goaf-filling technologies through technical standards, process supervision, and certified restoration while also committing to the development of detailed implementation rules and increased government financial support (for details, see Section S1). Encompassing both its successes and challenges, China’s experience in mine restoration is expected to serve as a crucial reference for other resource-dependent economies tackling integrated waste management.

**Figure 1:**
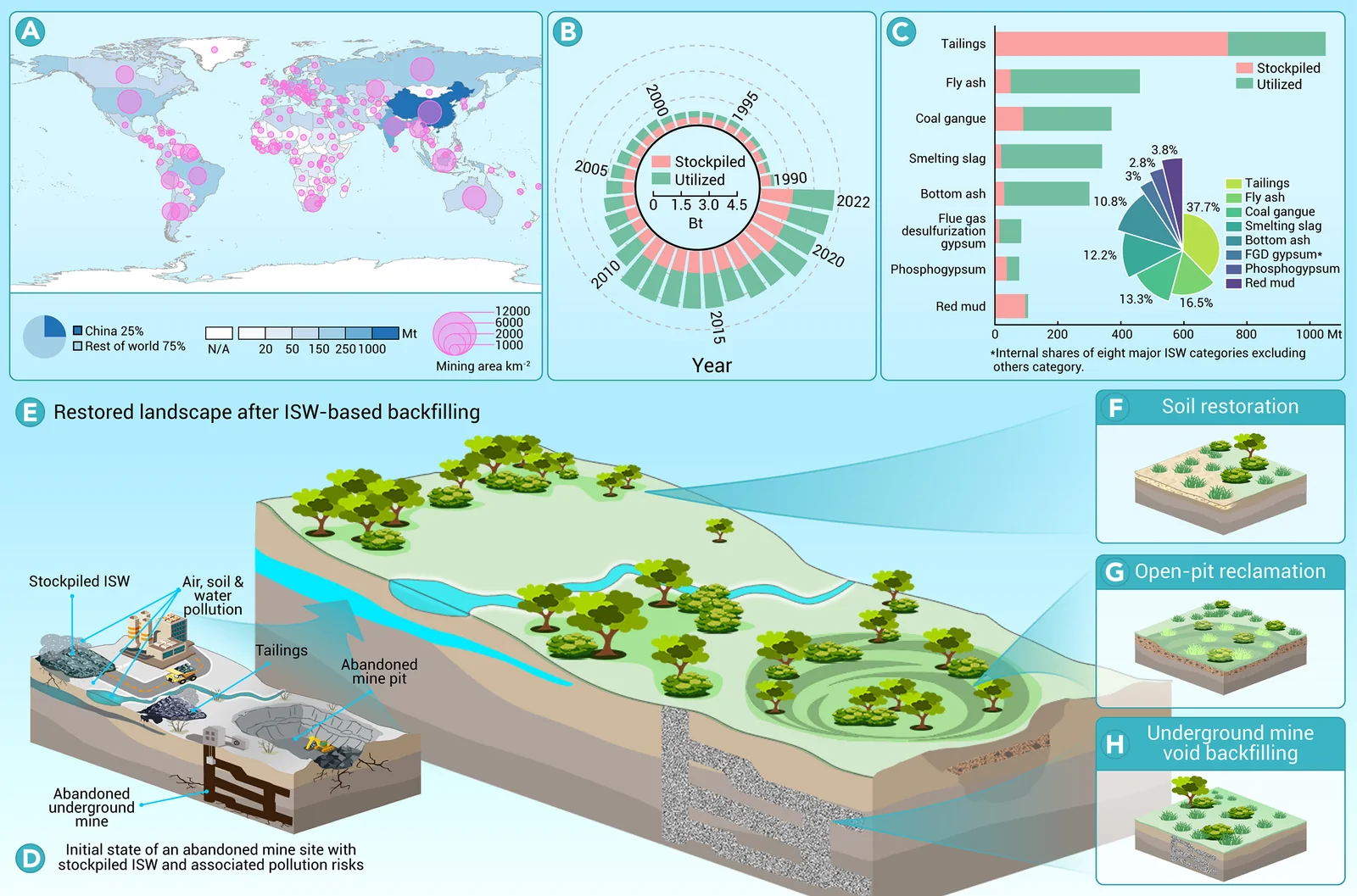
Potential and strategic approaches for industrial solid waste in the ecological restoration of abandoned mines (A) Geographic distribution of global ISW generation and associated mining areas in 2022. The color gradient represents the amount of ISW generated, bubble size indicates mining area, and the inset shows China’s share of global ISW generation in 2022 (Table S1; https://doi.org/10.1594/PANGAEA.942325). (B) Evolution of the annual increment in ISW stockpiling and the amount utilized in China from 1990 to 2022 (Table S2). (C) Generation of eight major categories of ISW in China in 2022, including the amounts utilized and stored. The inset shows the relative composition of these eight major ISW categories after excluding “other” categories, which accounted for 33.6% of total generation and were omitted for clarity; complete data are provided in Table S3. (D) Initial state of an abandoned mine site with stockpiled ISW and associated air, soil, and water pollution risks. (E) Restored landscape after ISW-based backfilling, including landform reconstruction, cover placement, and revegetation. (F–H) Specific restoration applications: (F) soil restoration through ISW-assisted soil conditioning and vegetation recovery; (G) open-pit reclamation through backfilling, topsoil placement, and revegetation; and (H) structural filling of underground mine voids. For data and methods, see the supplemental information (https://github.com/iPFAS/The-Innovation-ISW/tree/master).

## Rethinking the strategy for promising mine restoration by utilizing ISW

Mine restoration can reconnect industrial waste with the Earth’s material cycles while recovering landscapes shaped by decades of extraction. China has a large and growing stock of abandoned mines and disturbed mining landscapes (Figure 1A). A national survey by the China Geological Survey estimates that roughly 99,000 abandoned mines still require remediation.^2^ Demonstration projects led by natural-resources authorities indicate a shift from scattered pilots to scaled implementation; representative cases are presented in Table S4. Many abandoned pits and tailings areas are concentrated in mountainous and resource-dependent regions, often far from dense settlements. In administrative practice, they are readily framed as existing disturbed land that can be repurposed for waste accommodation alongside land reclamation. They also offer clear volumetric capacity, definable engineering boundaries for closure, and an evident need for landform reconstruction, making them an attractive setting for large-volume recovery uses of ISW. However, this pathway has not yet become a mainstream option. Its large-scale adoption is constrained by the prioritization of high-value resource recovery, the continued prevalence of simple and low-cost disposal methods, uncertainties regarding material suitability and engineering stability, and the potential long-term leaching and groundwater risks associated with direct mine-pit backfilling (see Section S2).

These pressures are global. Mining occupied 101,583 km^2^ of land by 2022 and increasingly encroaches on ecologically sensitive areas.^3^ Across China, Brazil, Canada, South Africa, Spain, the United States, and other countries, shared risk pathways include storage-facility instability, contaminant leaching, acidic or alkaline drainage, heavy-metal migration, and downstream water-quality degradation (Table S6). China is one representative case: 143 tailings storage facility failures between 1957 and 2023 caused 840 fatalities and released tens of millions of cubic meters of tailings slurry.^4^ Mine closures are also expanding the global restoration burden; by 2030, another 127 coal mines covering approximately 3,731 km^2^ are projected to close.^5^ Under these conditions, policy frameworks that integrate waste accommodation with the restoration of mining sites are becoming an increasingly attractive option for resource-dependent economies, offering a scalable pathway to address dual challenges.

Directing ISW into abandoned mine pits reuses already-degraded land and can limit the expansion of dedicated disposal sites. For local authorities, it combines waste management and land restoration within one operational setting. Engineered projects can reduce dust, slope instability, and erosion associated with open stockpiles and landfills (Figure 1D) while supplying bulk material for landform reconstruction (Figure 1E). Where material-qualification requirements and construction standards are effectively enforced, layered backfilling, compaction, capping, and revegetation can progressively transform degraded land into forests, grasslands, or other restored land uses (Figures 1F–1H), thereby shortening the duration of bare-land exposure and reducing persistent ecological disturbances caused by runoff and landscape fragmentation. The three pathways shown in Figures 1F–1H are not interchangeable and should be selected based on site conditions, material properties, ISW accommodation demand, and environmental risk controls (Section S3).

Fundamentally, this approach integrates ISW management and mine restoration within a single regulated project. Because it introduces external waste streams, it requires stricter whole-process oversight than conventional restoration, including material admission, engineering boundaries, environmental monitoring, and post-closure responsibilities (Section S4). Engineered backfilling should therefore not be treated as simple waste absorption but as a controlled restoration within defined boundaries, preventing informal dumping or dispersed stockpiling. Where high-value utilization is not currently viable, it can provide a manageable outlet while reducing the long-term safety burden of stockpiles and tailings facilities.

## Challenges of ISW-based mine restoration

Remoteness does not eliminate the risks of mine-pit backfilling. The central challenge is their delayed and spatially expanding character: in hydrologically connected mountainous catchments, contaminants can migrate through rainfall infiltration and groundwater transport, turning site-level hazards into cumulative basin-scale pressures. Releases may emerge only after waste weathering, backfill settlement, liner deterioration, or changes in pH and redox conditions, when remediation is harder and responsibilities are fragmented across mining, environmental, and land authorities. Institutionalized post-closure monitoring, secure funding, and transparent reporting are therefore essential to connect short construction cycles with long-term stewardship and preserve early-warning capacity.

Low-cost, high-capacity backfilling can also become a policy and market default, weakening incentives for higher-value recycling alternatives and raising the opportunity cost of alternative uses for mine voids. Regulatory gaps amplify this risk: without waste-flow tracking, interdepartmental coordination, effective approval, and post-closure accountability, landfill-like or poorly regulated backfilling may be easier than standardized recovery. Yet mine pits are increasingly considered for pumped-hydro storage and other energy infrastructure, while reclamation supports ecological restoration and carbon-sink strategies. Prematurely privileging backfilling can therefore foreclose future land-use options, constrain multi-objective planning, and erode long-term resource efficiency.

Data visibility is another governance challenge. Because primary ISW statistics remain incomplete, especially in developing countries, the global estimates presented here indicate broad patterns rather than provide a fully measured inventory. Fragmented reporting, inconsistent statistical boundaries and classifications, and uneven completeness may underestimate or mischaracterize pressures in data-scarce regions, distorting global assessments and policy priorities. Stronger primary data reporting, harmonized ISW classifications, and more transparent and comparable national statistics are therefore essential.

## Guidelines to promote mine restoration by utilizing ISW

For ISW-based mine restoration to constitute genuine recovery rather than disguised disposal, it must operate within transparent and enforceable policy guardrails. Regional waste plans should set explicit capacity ceilings and apply tiered, quota-based admission rules that prioritize higher-value recycling and reserve backfilling for residual streams lacking viable markets and are demonstrably low risk. Material admission must be matched to site conditions, considering leachability, long-term geochemical behavior, mechanical stability, and local hydrogeology. Waste rock, low-leaching non-metallic tailings, and verified inert wastes are generally suited to landform reconstruction in open pits, abandoned quarries, and sand pits. After pretreatment and risk assessment, coal gangue, fly ash, and furnace slag may support goaf filling, open-pit reclamation, and subsidence control; stabilized low-leaching tailings may be used for controlled backfilling or comparable tailings-facility remediation. Sulfide-rich, highly alkaline, saline, or metal-mobile wastes should be excluded unless stabilization, engineered containment, and long-term monitoring demonstrate controllable risk.

Once material-site compatibility is established, risk control must extend from pre-construction screening to post-closure stewardship. Hydrogeological assessment and material testing should precede backfilling; construction should combine low-permeability barriers and composite liners with leachate collection, runoff diversion, zoned placement, layered compaction, and surface capping. Post-closure monitoring should track groundwater, leachate, heavy metals, other mobile contaminants, settlement, and slope stability to detect delayed risks. Material provenance, composition, engineering implementation, and environmental performance should be disclosed through a public registry and independently audited, preventing waste disposal from being repackaged as ecological restoration.

Governance must span the full project life cycle. Environmental bonds, post-closure monitoring funds, or insurance mechanisms can formalize and finance long-term hydrological monitoring, risk response, and liability recovery, preventing obligations from ending with construction. Mine restoration must also be coordinated with competing land uses. Spatial plans should evaluate the long-term benefits and irreversible trade-offs among waste utilization, ecological recovery, energy infrastructure, and carbon sequestration. Where mine pits have multiple potential futures, phased and zoned strategies can preserve option value and prevent one-off engineering choices from locking in inefficient outcomes.

China’s mine-based ecological restoration policy illustrates how a state can open a recovery pathway when mounting waste pressures meet a large restoration backlog. By coupling spatial remediation with waste-flow management, mine restoration may relieve stockpiling and landfill pressures. Its success, however, will depend less on engineering feasibility than on institutions capable of maintaining environmental and circular-economy boundaries amid delayed risks, distorted incentives, and competing land uses. For other countries facing growing bulk ISW and extensive mining legacies, the central lesson is not simply to find new accommodation space but to build governance that keeps recovery measurable, enforceable, and subordinate to higher-value circular utilization.

## Funding and acknowledgments

This work was supported by the 10.13039/501100012166National Key Research and Development Program of China (no. 2024YFC3906402), the 10.13039/501100001809National Natural Science Foundation of China (no. 52470210), and the China Three Gorges Corporation Research Program (no. 202403033).

## Declaration of interests

The authors declare no competing interests.

## Supplemental information

Document S1. Tables S1–S6, Sections S1–S4, and supplemental methods

Document S2. Article plus supplemental information
