# Supplementary material for: Repurposing China’s industrial solid waste in mine ecological restoration
Source: Innovation (Camb). 2026 Sep 8;7(10):101567. doi: 10.1016/j.xinn.2026.101567 (PMC13673549; doi:10.1016/j.xinn.2026.101567)
Supplement: Supplementary file 1 [file mmc1.pdf]

The Innovation, Volume *7*

Supplemental Information

Repurposing China's industrial solid waste in mine ecological restoration

Qiuxia Zou, Ning Zhang, Zeguo Yang, Xiong Shi, Huabo Duan, Qifei Huang, and Jiakuan Yang

Supplementary Information:

Repurposing China's industrial solid waste in mine ecological restoration

Qiuxia Zou[1,2,6], Ning Zhang[3,6], Zeguo Yang[1,2], Xiong Shi[4], Huabo Duan[2]*, Qifei Huang[5]**, Jiakuan Yang[2]

[1] Institute of Artificial Intelligence, School of Artificial Intelligence and Automation, Huazhong University of Science and Technology, Wuhan 430074, China

[2] Hubei Key Laboratory of Multi-media Pollution Cooperative Control in Yangtze Basin, School of Environmental Science & Engineering, Huazhong University of Science and Technology, Wuhan 430074, China

[3] Department of Civil and Environmental Engineering, University of California, Davis, CA 95616, USA

[4] National Engineering Research Center of Eco-Environment in the Yangtze River Economic Belt, Wuhan 430014, China

[5] State Environmental Protection Key Laboratory of Hazardous Waste Identification and Risk Control, Chinese Research Academy of Environmental Sciences, Beijing 100012, China

[6] These authors contributed equally

Correspondence: huabo@hust.edu.cn (HDuan); huangqf@craes.org.cn (QHuang).

Table list and data source

Sheet name	Content	Data Source
Table S1-S3	Table S1 Global Industrial Waste Generation Data in 2022	• China: Ministry of Ecology and Environment of the People's Republic of China annual reports.<br>• Japan: Ministry of the Environment, Government of Japan annual reports.<br>• EU: Eurostat industrial waste databases (EU-27 member states)[1].<br>• United States: Estimated using IW generation intensity factor derived from Krones[2] and Chen et al[3].
	Table S2 Net increase in stockpiles, utilization rate, and cumulative stockpiles of industrial solid waste in China from 1990 to 2022	China Statistical Yearbook
	Table S3 ISW category generation, annual net inventory growth, and utilization in China in 2022	2022 Annual Report on the Prevention and Control of Environmental Pollution from Solid Waste in Large and Medium-Sized Cities Nationwide<br>Phosphogypsum[4]<br>Red mud[5]
Table S4	Table S4. Typical cases of mine restoration using industrial solid waste in China	
Table S5	Table S5 Correlation test results between AREA and ISW	
Table S6	Table S6 Representative examples of long-term environmental risks associated with mining waste and bulk industrial solid waste worldwide	
Methods	Global industrial solid waste generation projections and correlation analysis methods with mining area	

Table S1 Global Industrial Waste Generation Data in 2022（Million tonnes）

Year	Country Name	ISW	Flag	Year	Country Name	ISW	Flag
2022	Albania	1.2	collected	2022	Latvia	2.7	collected
2022	Algeria	96.0	estimated	2022	Lesotho	2.8	estimated
2022	Angola	99.5	estimated	2022	Liberia	3.7	estimated
2022	Argentina	125.8	estimated	2022	Libya	120.3	estimated
2022	Armenia	2.7	estimated	2022	Lithuania	5.8	collected
2022	Aruba	0.6	estimated	2022	Luxembourg	3.2	collected
2022	Australia	112.8	estimated	2022	Madagascar	61.3	estimated
2022	Austria	21.0	collected	2022	Malawi	81.7	estimated
2022	Azerbaijan	27.2	estimated	2022	Malaysia	108.4	estimated
2022	Bahamas	0.6	estimated	2022	Maldives	0.8	estimated
2022	Bahrain	6.8	estimated	2022	Mali	65.1	estimated
2022	Bangladesh	201.7	estimated	2022	Malta	0.6	collected
2022	Barbados	0.7	estimated	2022	Mauritania	5.7	estimated
2022	Belarus	59.2	estimated	2022	Mauritius	1.9	estimated
2022	Belgium	55.0	collected	2022	Mexico	153.2	estimated
2022	Belize	0.6	estimated	2022	Moldova	1.7	estimated
2022	Benin	54.1	estimated	2022	Mongolia	5.6	estimated
2022	Bolivia	29.0	estimated	2022	Montenegro	0.8	collected
2022	Bosnia and Herzegovina	7.4	collected	2022	Morocco	118.7	estimated
2022	Botswana	3.4	estimated	2022	Mozambique	56.5	estimated
2022	Brazil	290.4	estimated	2022	Myanmar	152.5	estimated
2022	Brunei Darussalam	0.8	estimated	2022	Namibia	2.2	estimated
2022	Bulgaria	101.7	collected	2022	Nepal	106.9	estimated
2022	Burkina Faso	64.1	estimated	2022	Netherlands	51.9	collected
2022	Burundi	30.2	estimated	2022	New Zealand	27.1	estimated
2022	Cabo Verde	0.6	estimated	2022	Nicaragua	24.1	estimated
2022	Cambodia	46.8	estimated	2022	Niger	67.4	estimated
2022	Cameroon	86.9	estimated	2022	Nigeria	154.2	estimated
2022	Canada	96.3	estimated	2022	North Macedonia	2.0	collected
2022	Central African Republic	20.2	estimated	2022	Norway	9.3	collected
2022	Chad	31.1	estimated	2022	Oman	15.2	estimated
2022	Chile	141.4	estimated	2022	Pakistan	147.0	estimated
2022	China	4,113.7	collected	2022	Panama	10.1	estimated
2022	Colombia	167.5	estimated	2022	Papua New Guinea	33.6	estimated
2022	Comoros	0.8	estimated	2022	Paraguay	27.9	estimated
2022	Congo, Dem. Rep.	125.1	estimated	2022	Peru	127.4	estimated
2022	Costa Rica	18.1	estimated	2022	Philippines	208.1	estimated
2022	Croatia	5.4	collected	2022	Poland	188.9	collected

Year	Country Name	ISW	Flag	Year	Country Name	ISW	Flag
2022	Cyprus	2.0	collected	2022	Portugal	17.4	collected
2022	Czechia	27.6	collected	2022	Puerto Rico	6.0	estimated
2022	Denmark	7.6	collected	2022	Qatar	8.0	estimated
2022	Djibouti	0.9	estimated	2022	Romania	163.9	collected
2022	Dominican Republic	70.3	estimated	2022	Russian Federation	183.5	estimated
2022	Ecuador	59.6	estimated	2022	Rwanda	32.2	estimated
2022	Egypt, Arab Rep.	167.6	estimated	2022	Samoa	0.6	estimated
2022	El Salvador	35.4	estimated	2022	Sao Tome and Principe	0.7	estimated
2022	Equatorial Guinea	5.4	estimated	2022	Saudi Arabia	128.9	estimated
2022	Estonia	24.5	collected	2022	Senegal	35.7	estimated
2022	Eswatini	1.0	estimated	2022	Serbia	52.8	estimated
2022	Ethiopia	105.6	estimated	2022	Seychelles	0.5	estimated
2022	Fiji	0.9	estimated	2022	Sierra Leone	27.3	estimated
2022	Finland	110.0	collected	2022	Slovak Republic	13.9	collected
2022	France	105.7	collected	2022	Slovenia	6.6	estimated
2022	Gabon	4.4	estimated	2022	Solomon Islands	0.7	estimated
2022	Georgia	5.0	estimated	2022	Somalia	28.2	estimated
2022	Germany	241.9	collected	2022	South Africa	144.8	estimated
2022	Ghana	110.4	estimated	2022	Spain	89.9	collected
2022	Greece	23.1	collected	2022	Sri Lanka	161.7	estimated
2022	Grenada	0.6	estimated	2022	St. Lucia	0.7	estimated
2022	Guatemala	54.6	estimated	2022	St. Vincent and the Grenadines	0.6	estimated
2022	Guinea	41.2	estimated	2022	Sudan	107.1	estimated
2022	Guinea-Bissau	3.1	estimated	2022	Suriname	0.7	estimated
2022	Guyana	1.2	estimated	2022	Sweden	150.7	collected
2022	Haiti	23.9	estimated	2022	Switzerland	48.5	estimated
2022	Honduras	28.4	estimated	2022	Tajikistan	31.1	estimated
2022	Hungary	17.4	collected	2022	Tanzania	97.7	estimated
2022	Iceland	0.7	estimated	2022	Thailand	239.6	estimated
2022	India	2,670.6	estimated	2022	Timor-Leste	3.1	estimated
2022	Indonesia	306.7	estimated	2022	Togo	29.0	estimated
2022	Iran, Islamic Rep.	120.6	estimated	2022	Trinidad and Tobago	6.6	estimated
2022	Iraq	92.0	estimated	2022	Tunisia	35.1	estimated
2022	Ireland	17.2	collected	2022	Turkiye	104.9	collected
2022	Israel	46.4	estimated	2022	Turkmenistan	35.1	estimated
2022	Italy	148.5	collected	2022	Uganda	102.5	estimated
2022	Jamaica	1.9	estimated	2022	Ukraine	151.1	estimated
2022	Japan	232.5	estimated	2022	United Arab Emirates	79.8	estimated
2022	Jordan	58.7	estimated	2022	United Kingdom	169.1	estimated
2022	Kazakhstan	82.5	estimated	2022	United States	181.0	estimated
2022	Kenya	107.4	estimated	2022	Uruguay	10.3	estimated

Year	Country Name	ISW	Flag	Year	Country Name	ISW	Flag
2022	Kiribati	0.6	estimated	2022	Uzbekistan	124.0	estimated
2022	Korea, Rep.	135.3	estimated	2022	Vanuatu	0.6	estimated
2022	Kyrgyz Republic	34.5	estimated	2022	Zambia	61.0	estimated
2022	Lao PDR	41.0	estimated	2022	Zimbabwe	60.4	estimated

Note: “collected” data were derived from official statistics and published literature, whereas “estimated” data were estimated using machine-learning models. Detailed methods are provided in the Supplementary Methods section.

Table S2 Net increase in stockpiles, utilization rate, and cumulative stockpiles of industrial solid waste in China from 1990 to 2022

Year	Stockpiled	Utilized	Cumulative stockpiles
	Billion tonnes		
1990	0.57	0.17	4.38
1991	0.37	0.22	4.75
1992	0.41	0.26	5.16
1993	0.42	0.25	5.58
1994	0.42	0.27	6.01
1995	0.39	0.29	6.40
1996	0.38	0.28	6.78
1997	0.39	0.30	7.17
1998	0.38	0.33	7.55
1999	0.37	0.36	7.92
2000	0.38	0.37	8.30
2001	0.45	0.47	8.74
2002	0.47	0.50	9.21
2003	0.45	0.56	9.67
2004	0.53	0.68	10.19
2005	0.59	0.77	10.78
2006	0.65	0.93	11.44
2007	0.65	1.10	12.09
2008	0.70	1.23	12.79
2009	0.68	1.38	13.48
2010	0.81	1.62	14.29
2011	1.31	1.95	15.60
2012	1.31	2.02	16.90
2013	1.26	2.06	18.16
2014	1.25	2.04	19.41
2015	1.31	1.99	20.73
2016	1.28	1.84	22.01
2017	1.58	1.81	23.59
2018	1.58	1.81	25.17
2019	1.87	2.06	27.05
2020	1.73	2.04	28.77
2021	1.78	2.27	30.55
2022	1.83	2.37	32.38

Note: Data are derived from the China Statistical Yearbook (1983–2023)[6]. The industrial solid waste categories covered include tailings, fly ash, coal gangue, smelting slag, and other industrial solid wastes.

Table S3 ISW category generation, annual net inventory growth, and utilization in China in 2022

ISW category	Generation	Utilized	Stockpiled
	Million tonnes		
Tailings	1050	310	740
Fly ash	460	410	50
Coal gangue	370	280	90
Smelting slag	340	320	20
Bottom ash	300	270	30
Flue gas desulfurization gypsum gypsum	84	69	15
Phosphogypsum	77	39	38
Red mud	105	8	97
Others	1411		

Table S4. Typical cases of mine restoration using industrial solid waste in China

Project	Location	Solid waste type	Utilization amount (Mt)	Application target	Source
Ecological restoration project of abandoned sand pits and open-pit mines in Baotou City	Baotou, Inner Mongolia	General industrial solid wastes, including tailings, fly ash, slag, etc.	>10.0 (2021–2023)	Abandoned sand pits and abandoned open-pit mines	[7]
Dushan abandoned mine pit ecological restoration and general industrial solid waste disposal project	Tongling, Anhui	General industrial solid waste	6.64 (2018–2020)	Abandoned mine pit	[8]
Underground intelligent gangue sorting and goaf grouting disposal project of Huangling Mining No. 2 Coal Mine	Huangling, Yan'an, Shaanxi	Coal gangue	0.20	Underground goaf of coal mine	[9]
Green backfill mining project of Zaozhuang Mining Group	Jining, Shandong / Zaozhuang Mining Group	Coal gangue, fly ash, slag, etc.	0.7	Underground goaf	[10]
Ecological restoration case of Heidaigou Open-pit Coal Mine, Guoneng Zhungeer Energy Group Co., Ltd.	Zhungeer Banner, Ordos, Inner Mongolia	Coal gangue and coal-based solid wastes	N/A	Mine pits, goafs, and damaged lands	[11]
Ecological reclamation project using coal gangue at Bulianta Coal Mine, Shendong Coal, National Energy Group	Ordos, Inner Mongolia	Coal gangue	11.0	Abandoned mining areas, open-pit mine pits, subsidence areas, natural gullies, and other reclamation sites	[12]

Notes:1) Mt: million tonnes; 2) Goaf refers to underground mined-out areas left after coal extraction; 3) N/A indicates that the publicly available source did not report a specific utilization quantity.

Table S5 Correlation test results between AREA and ISW

Method	Coefficient	p-value
Pearson (raw scale)	0.499	1.36×10^{-9}
Spearman (rank)	0.504	8.22×10^{-10}
Pearson (log–log)	0.527	1.04×10^{-10}

Table S6 Representative examples of long-term environmental risks associated with mining waste and bulk industrial solid waste worldwide

Industrial solid waste type	Country/Region	Year	Primary long-term risks	References
Tailings	China	1957-2023	Tailings dam instability, catastrophic release of tailings, heavy metal leaching, groundwater and surface water contamination, ecosystem degradation, and persistent environmental liabilities from inactive or abandoned tailings storage facilities.	13
	Brumadinho, Brazil	2019	Long-term pollution of river and floodplain sediments; Resuspension of heavy metals; Ecological and human exposure risks at the watershed scale.	14
	Mariana, Brazil	2015	Persistent metal contamination in river sediments, bioaccumulation in aquatic organisms, cytotoxic and genotoxic effects, and long-term degradation of riverine, estuarine, and coastal ecosystems	15
	Mount Polley, Canada	2014	Declining geochemical stability of sediments; Long-term effects of secondary disturbance on metal migration and clearance processes.	16
	Soweto, South Africa	-	Combined exposure to radiation and chemical toxicity; Health risks to residents and	17

Industrial solid waste type	Country/Region	Year	Primary long-term risks	References
			risks associated with land reuse.	
	Aznalcóllar, Spain	1998	Acidic mine wastewater continues to be generated; Soil and sediments exhibit long-term heavy metal accumulation.	18
Fly ash	Kingston, USA	2008	Long-term release and redistribution of trace metals in river sediments.	19
	Aligarh, India	-	Heavy metal contamination of soil and groundwater; Chronic exposure risk.	20
Coal gangue	Upper Silesia, Poland	-	Persistent self-heating and spontaneous combustion; long-term heavy-metal mobilization and acid mine drainage; accumulation and release of PAHs and chlorinated persistent organic pollutants; toxic gaseous emissions; and elevated lifetime carcinogenic risks to nearby populations.	21
Slag	Miedziana Góra, Poland	-	Centuries-long soil contamination by Pb, Cu, Zn, As, and other metal(loid)s; pH-dependent pollutant remobilization; continued phytoavailable metal release; and persistent Pb contamination of soil pore water.	22
Phosphogypsum	Florida, USA	-	Recurrent wastewater releases from aging phosphogypsum stacks; persistent nutrient enrichment and eutrophication; harmful algal blooms, declining water quality, fish mortality, and potential long-term degradation of seagrass and estuarine ecosystems.	23

Industrial solid waste type	Country/Region	Year	Primary long-term risks	References
Red mud	Ajka, Hungary	2010	Long-term soil salinization; persistent leaching and cycling of mobile metals and metalloids, particularly V, Mo, As, Cu, and Ni, in soil–water systems; potential phosphorus-driven eutrophication; and chronic ecotoxicological effects.	[24]

Section 1

Management requirements for mine backfilling and goaf filling. More specifically, Item (11) of the *Action Plan for Comprehensive Solid Waste Management* calls for the "prudent and orderly exploration of large-scale utilization pathways." Provided that environmental quality standards, pollution-risk-control requirements, and work-safety requirements are satisfied, the policy encourages the large-scale utilization of bulk industrial solid waste through underground filling, mine-pit backfilling, ecological restoration, and other approaches. At the same time, it requires the establishment of a unified and standardized management system, stronger interdepartmental coordination, and strict compliance with the relevant approval and decision-making procedures to prevent illegal dumping under the guise of large-scale utilization. Therefore, rather than regulating mine backfilling and goaf-filling technologies by prescribing specific engineering parameters, the *Action Plan for Comprehensive Solid Waste Management* incorporates them into controlled pathways for resource utilization and ecological restoration by defining their applicable conditions, institutional boundaries, and regulatory safeguards.

Section 2

Constraints on the large-scale application of industrial solid waste in mine ecological restoration

The use of industrial solid waste in mine ecological restoration has not yet become a mainstream pathway, mainly because of the combined constraints of policy orientation, economic cost, technical compatibility, and environmental risk. First, in terms of policy orientation, existing solid-waste resource-utilization policies generally prioritize high-value applications, such as the production of construction materials and recycled products, as well as raw-material substitution. By contrast, mine-pit backfilling and ecological restoration are more applicable to residual solid wastes that lack market demand or are difficult to utilize through high-value pathways; consequently, their policy priority and market attractiveness remain relatively limited.

Second, simple and low-cost landfilling or stockpiling practices persist in some regions, reducing the incentives for enterprises and local governments to invest in the more costly processes of material pretreatment, engineering design, and long-term monitoring. Third, uncertainties remain regarding material suitability and engineering stability. Different types of industrial solid waste vary substantially in particle-size distribution, mechanical strength, compaction performance, cementitious properties, and long-term stability. Without rigorous material screening and engineering adaptation, these differences may compromise the stability of the backfill body, slope safety, and the recovery of post-restoration land functions.

Fourth, direct mine-pit backfilling may also pose long-term leaching and groundwater-contamination risks. Heavy metals, salts, and other mobile constituents in some solid wastes may be continuously released under rainfall infiltration and groundwater movement, allowing contamination to spread from an individual mine pit to broader groundwater systems or river basins. Therefore, although the use of industrial solid waste in mine ecological restoration has considerable potential for large-scale waste absorption and land rehabilitation, its wider application must be based on material-admission criteria, engineering controls, and long-term environmental risk management.

Section 3

Comparison of ISW-based mine restoration pathways

Soil remediation primarily targets surface-soil reconstruction and vegetation recovery and therefore has a relatively limited absorption capacity. Mine-pit reclamation is applicable to abandoned open-pit mines, quarries, sand pits, and similar sites, and generally offers greater volumetric absorption potential because the pits themselves provide clearly defined engineering boundaries and substantial spatial capacity. Underground restoration or goaf filling is applicable to underground coal mines and certain metal mines. Paste or slurry backfilling can reduce surface stockpiling and control land subsidence, but its feasibility is constrained by goaf geometry, backfill strength, mine safety, transportation systems, and groundwater conditions. The three pathways are therefore not interchangeable; rather, they should be matched according to site morphology, material properties, waste-absorption requirements, and environmental risk-control conditions, while collectively contributing to shorter periods of bare-land exposure, reduced runoff disturbance, and the restoration of mining landscapes.

Section 4

Differences between conventional mine ecological restoration and ISW-based backfilling restoration

Conventional mine ecological restoration and ISW-based backfilling restoration both aim to restore the land and ecological functions of disturbed mining areas, but they differ substantially in regulatory objects, risk sources, and full-process management requirements. Conventional mine ecological restoration mainly focuses on landform reshaping, slope stabilization, soil reconstruction, topsoil placement, revegetation, and post-restoration maintenance. Its supervision is primarily concerned with restoration quality, land reclamation performance, and ecological recovery outcomes.

By contrast, ISW-based backfilling introduces external solid-waste streams into the restoration process. Therefore, in addition to meeting ecological restoration requirements, it must also comply with solid-waste environmental management requirements. Its supervision should cover not only restoration outcomes, but also waste provenance, waste category, material eligibility, pretreatment, transportation records, zoned backfilling, leachate and groundwater monitoring, and post-closure responsibility.

Therefore, ISW-based backfilling restoration should not be treated as a simple variant of conventional mine ecological restoration. Rather, it should be governed as a coupled “solid-waste management–mine ecological restoration” project. The core regulatory objective is to ensure that the entire solid-waste utilization process is identifiable, traceable, and auditable, thereby preventing ecological restoration from being misused as disguised disposal or informal dumping.

Supporting methods

Global industrial solid waste projection. The industrial solid waste (ISW) prediction project follows a structured machine learning workflow. (Please refer to the open source code: https://github.com/iPFAS/global_solid_waste_prediction)

Our approach to predicting ISW generation involved several key steps. First, we gathered and meticulously cleaned historical data on waste generation along with relevant socioeconomic factors for numerous countries, including economic indicators (like GDP per capita adjusted for purchasing power), population figures, regional classifications, and income groupings. This prepared dataset formed the foundation for our analysis.

Next, we derived a set of more informative predictive factors from this base data. These factors were designed to capture underlying trends related to economic development stages, population dynamics, urbanization patterns, and changes over time, providing richer input for the prediction models. The waste generation figures themselves were also mathematically adjusted to help improve the models' predictive capabilities.

To ensure the reliability and generalizability of our predictions, we employed a rigorous validation strategy. The data was carefully divided to test the model in two critical ways: predicting waste generation for recent years the model hadn't seen before (temporal validation) and predicting for entire countries that were not included during the model building phase (geographical validation). This dual approach assesses the model's ability to forecast future trends and its applicability to new contexts.

We then developed the predictive models using established statistical and machine learning techniques. Multiple modeling approaches were evaluated based on their performance during the validation process. The strengths of the most successful methods were combined into a final, more accurate and robust composite model.

Finally, the predictive accuracy of this final model was thoroughly assessed using the reserved test data, employing standard statistical measures to quantify its performance.

Correlation relationship between mining area and industrial solid waste. To examine the statistical association between the scale of mining activities (AREA) and the magnitude of ISW generation at the country level, we conducted a dual test using Pearson's correlation coefficient and Spearman's rank correlation coefficient.

Data processing is performed at the country level. Two indicators—AREA and ISW—are collected. After excluding observations with missing values in either variable, a total of 131 countries are retained in the final sample.

Given that AREA and ISW exhibit pronounced right-skewness and span multiple orders of magnitude across countries, Spearman rank correlation is used as the primary measure to mitigate the undue

influence of a small number of extreme values on linear correlation estimates, while Pearson correlations (on the raw scale and in log–log form) are reported as complementary evidence. All p-values are based on two-sided tests. Importantly, correlation captures statistical association only and should not be interpreted as causal evidence.

Additional notes. We recognize that definitions of industrial waste may vary across countries and institutions, with potential gaps in coverage. This paper defines industrial waste as solid waste generated during industrial production and processing that does not qualify as hazardous waste and requires collection, management, and disposal (primarily referring to general industrial solid waste). Classification is based on official or authoritative databases from various countries, with corresponding categorization and consolidation to support macro-level trend identification rather than precise statistical measurement.

The Gross Domestic Product based on purchasing power parity (GDP PPP, constant 2017 international $) and GDP PPP per capita (constant 2017 international $) series used in this paper are drawn from the World Bank's World Development Indicators (WDI) and follow the rationale in Hoy et al. (2023)[1] in preferring PPP-adjusted measures over standard GDP and GDP per capita for cross-country comparability.

The procedures for assembling the socioeconomic input dataset and defining the prediction coverage—limited to country-years with the required WDI inputs and excluding cases with missing WDI data—are documented in the WDI metadata and described in Hoy et al. (2023)[25].

References

1. European Commission, "Generation of waste by waste category, hazardousness and NACE Rev. 2 activity" (Eurostat, 2024); https://ec.europa.eu/eurostat/databrowser/view/env_wasgen__custom_12171597/default/table?lang=en.
2. Krones JS. Accounting for non-hazardous industrial waste in the United States 2016; Doctoral dissertation, Massachusetts Institute of Technology.
3. Chen J, Li X, Huang K, Eckelman MJ, Chertow MR, Jiang D. Non-hazardous industrial waste in the United States: 100 Million tonnes of recoverable resources. Resources, Conservation and Recycling 167, 105369 (2021). https://doi.org/https://doi.org/10.1016/j.resconrec.2020.105369.
4. Shi, X., Zeng, A., Duan, H., Zhang, H. & Yang, J. Status and development trends of phosphogypsum utilization in China. Circular Economy 3, 100116 (2024). https://doi.org/https://doi.org/10.1016/j.cec.2024.100116.
5. Li, Q., Geng, C., Zhang, H., Shi, X., Liu, J. & Chen, C. The large-scale sustainable utilization status of bauxite residue (red mud): challenges and perspectives for China. Environmental Reviews 33, 1 – 16 (2025). https://doi.org/https://doi.org/10.1139/er-2024-0007

6. National Bureau of Statistics of People’s Republic of China. China Statistical Yearbooks. China Statistics Press, 1983-2023.
7. Han, W., Zhang, C.Q., Wang, H.Y., et al. Progress, problems and countermeasures of bulk industrial solid waste for ecological restoration of abandoned open pits. Environmental Protection Science 51, 20-24 (2025). https://doi.org/10.16803/j.cnki.issn.1004-6216.202403047. (In Chinese)
8. Ministry of Ecology and Environment of the People's Republic of China. Tongling City's pilot "Zero-waste City" construction: Highlighted practices (2020). https://www.mee.gov.cn/home/ztbd/2020/wfcsjssdgz/sdjz/ldms/202006/t20200601_782123.shtml.
9. Tencent News. Coal gangue backfilling as a pathway for green and low-carbon development of Huangling Mining No. 2 Coal Mine (2022). https://news.qq.com/rain/a/20220816A0619V00.
10. Shandong Energy Zaozhuang Mining Group. Green backfill mining project using coal gangue and other industrial solid wastes (2024). https://zzky.shandong-energy.com/185709/185711/2024/12/36685483.html.
11. China Coal News. Coal gangue ecological reclamation practice of Bulianta Coal Mine (2025). https://epaper.ceic.com/pad/content/202504/11/content_12648.html.
12. China Energy Investment Corporation. Ecological restoration practices using coal gangue at Heidaigou Open-pit Coal Mine (2025). https://www.ceic.com/gjnyjtww/chnyxfc/202505/cbbe5e06c64240da9b8f48e704d714b3.shtml.
13. Su, C., Rana, N.M., Evans, S.G., Wang, B., and Zhang, S. Tailings storage facilities in China: Historical failure incidents, existing status, and database-driven quantitative risk assessment. International Journal of Disaster Risk Reduction 114, 104973 (2024). https://doi.org/10.1016/j.ijdrr.2024.104973.
14. Silva Rotta, L.H., Alcântara, E., Park, E., Negri, R.G., Lin, Y.N., Bernardo, N., Mendes, T.S.G. & Souza Filho, C.R. The 2019 Brumadinho tailings dam collapse: Possible cause and impacts of the worst human and environmental disaster in Brazil. International Journal of Applied Earth Observation and Geoinformation 90, 102119 (2020). https://www.sciencedirect.com/science/article/pii/S0303243420300192.
15. Vergilio, C.d.S., Lacerda, D., Souza, T.d.S., et al. Immediate and long-term impacts of one of the worst mining tailing dam failures worldwide (Bento Rodrigues, Minas Gerais, Brazil). Science of the Total Environment 756, 143697 (2021). https://doi.org/10.1016/j.scitotenv.2020.143697.
16. Bird, G., Hudson-Edwards, K.A., Byrne, P., Macklin, M.G., Brewer, P.A. & Williams, R.D. River sediment geochemistry and provenance following the Mount Polley mine tailings spill, Canada: The role of hydraulic sorting and sediment dilution processes in contaminant dispersal and remediation. 134, 105086 (2021). https://www.sciencedirect.com/science/article/pii/S0883292721002171.
17. Zupunski, L., Street, R., Ostroumova, E., Winde, F., Sachs, S., Geipel, G., Nkosi, V., Bouaoun, L., Haman, T., Schüz, J. & Mathee, A. Environmental exposure to uranium in a population living in close proximity to gold mine tailings in South Africa. Journal of Trace Elements in Medicine

and Biology 77, 127141 (2023). https://www.sciencedirect.com/science/article/pii/S0946672X23000172.

18. Kraus, U. & Wiegand, J. Long-term effects of the Aznalcóllar mine spill—heavy metal content and mobility in soils and sediments of the Guadiamar river valley (SW Spain). Science of The Total Environment 367(2), 855–871 (2006). https://www.sciencedirect.com/science/article/pii/S0048969705009253.

19. Ramsey, A.B., Faiia, A.M. & Szynkiewicz, A. Eight years after the coal ash spill – Fate of trace metals in the contaminated river sediments near Kingston, eastern Tennessee. Applied Geochemistry 104, 158–167 (2019). https://www.sciencedirect.com/science/article/pii/S0883292719300630.

20. Khan, I. & Umar, R. Environmental risk assessment of coal fly ash on soil and groundwater quality, Aligarh, India. Groundwater for Sustainable Development 8, 346–357 (2019). https://www.sciencedirect.com/science/article/pii/S2352801X18301036.

21. Nádudvari, Á., Kozielska, B., Abramowicz, A., Fabiańska, M., Ciesielczuk, J., Cabała, J. & Krzykawski, T. Heavy metal-and organic-matter pollution due to self-heating coal-waste dumps in the Upper Silesian Coal Basin (Poland). Journal of Hazardous Materials 412, 125244 (2021). https://doi.org/10.1016/j.jhazmat.2021.125244.

22. Kupczak, K., Warchulski, R., Ettler, V. & Mihaljevič, M. The impact of buried historical copper slags on contemporary soil contamination. Journal of Geochemical Exploration 273, 107743 (2025). https://doi.org/10.1016/j.gexplo.2025.107743.

23. Beck, M.W., Altieri, A., Angelini, C., Burke, M.C., Chen, J., Chin, D.W., Gardiner, J., Hu, C., Hubbard, K.A., Liu, Y., Lopez, C., Medina, M., Morrison, E., Phlips, E.J., Raulerson, G.E., Scolaro, S., Sherwood, E.T., Tomasko, D., Weisberg, R.H. & Whalen, J. Initial estuarine response to inorganic nutrient inputs from a legacy mining facility adjacent to Tampa Bay, Florida. Marine Pollution Bulletin 178, 113598 (2022). https://doi.org/10.1016/j.marpolbul.2022.113598.

24. Mayes, W.M., Burke, I.T., Gomes, H.I., Anton, Á.D., Molnár, M., Feigl, V. & Ujaczki, É. Advances in Understanding Environmental Risks of Red Mud After the Ajka Spill, Hungary. Journal of Sustainable Metallurgy 2(4), 332–343 (2016). https://doi.org/10.1007/s40831-016-0050-z

25. Hoy ZX, Woon KS, Chin WC, Van Fan Y, Yoo SJ. Curbing global solid waste emissions toward net-zero warming futures. Science 2023; 382(6672):797-800. https://doi.org/10.1126/science.adg3177.
